# Health-Related Quality of Life and Mental Health of Adolescents Involved in School Bullying and Homophobic Verbal Content Bullying

**DOI:** 10.3390/ijerph16142622

**Published:** 2019-07-23

**Authors:** Natalia Albaladejo-Blázquez, Rosario Ferrer-Cascales, Nicolás Ruiz-Robledillo, Miriam Sánchez-SanSegundo, Manuel Fernández-Alcántara, Elisa Delvecchio, Juan Carlos Arango-Lasprilla

**Affiliations:** 1 Department of Health Psychology, Faculty of Health Sciences, University of Alicante, 03690 Alicante, Spain; 2 Department of Philosophy, Social Sciences and Education; Università degli Studi di Perugia, 06123 Perugia, Italy; 3 BioCruces Bizkaia Health Research Institute, Cruces University Hospital Barakaldo, 48903 Bizkaia, Spain; 4 IKERBASQUE, Basque Foundation for Science, 48013 Bilbao, Spain; 5 Department of Cell Biology and Histology, University of the Basque Country UPV/EHU, 48940 Leioa, Spain

**Keywords:** bullying, homophobic verbal content bullying, homophobic name-calling, Health-Related Quality of Life, protective factors, adolescents

## Abstract

Bullying has been traditionally related to a significant reduction in well-being and Health-Related Quality of Life (HRQoL) of adolescents. This negative impact on HRQoL seems to be modulated by the developed role in bullying (uninvolved, bully, victim or bully-victim). However, no studies have identified if these negative results are the same when other types of bullying, such as homophobic bullying, are evaluated. The main aim of the present study was to analyze the prevalence of different roles of bullying and homophobic bullying and the relationship between these roles in both types of bullying with HRQoL, depression and anxiety levels in a sample of 1723 Spanish adolescents. Although results exhibited lower prevalence of homophobic bullying roles when compared to traditionally bullying in general, in the case of victims, the prevalence was high in the case of homophobic bullying. When differences between roles in HRQoL, depression and anxiety were evaluated, in both types of bullying, uninvolved adolescents showed the best results and bully-victim adolescents the worst. The obtained results suppose an improvement in the understanding of the negative effects of different types of bullying on HRQoL and mental health in adolescents. Future research could advance in this comprehension, analyzing possible differences with other types of bullying, such as cyberbullying.

## 1. Introduction

Bullying is defined as aggressive, unjustified, intentional and persistent behavior, characterized by the power imbalance between victim and aggressor [1,2] with negative psychological and social consequences for the lives of children and adolescents [3]. Its prevalence varies in different studies between 10 and 50% in adolescents [3,4,5]. In terms of sex differences in victimization, various investigations attribute greater protagonism to boys [6,7]; in contrast, other works highlight that females are more frequently victims of bullying than males [8,9], whereas other studies find no sex differences in victimization [10,11]. Given the inconsistent results regarding sex differences, it is necessary to describe these differences according to the role played in different types of school violence.

Previous research indicates that a large number of young people who are the target of bullying belong to minority groups or groups that are socially stigmatized due to their sexual orientation and gender identity [12,13]. In this sense, homophobic bullying is considered a form of bullying that is directed at people because of their sexual orientation and/or gender identity, either perceived or real [14]. This type of behavior ranges from social exclusion or rejection to physical or verbal violence and often includes mockery, homophobic insults, derision, nicknames, and intimidation [15,16].

The prevalence of homophobic bullying is relevant in all countries worldwide and in all social classes, as evidenced by previous studies in the USA and Europe in which it has been estimated that between 45 and 92% of lesbian, gay, bisexual, and transgender (LGBT) youth had been victims of homophobic insults [17,18]. In Spain, sexual minorities are also at higher risk of suffering bullying [19]. This high prevalence has also been corroborated in recent studies in non-heterosexual youth highlighting the global nature of homophobic bullying [20,21].

Homophobia is one of the main reasons for insulting, mocking, and rejecting classmates at school in Europe and is expressed through the use of homophobic language [22,23]. The use of this language goes far beyond sexual orientation and is also aimed at heterosexual youth [24,25], so it must be taken into account that, whether or not an individual belongs to a sexual minority, the existence of being labeled by the group as different is enough to initiate violent action.

The use of homophobic insults progressively increases from primary to secondary school, an educational stage where there is a high relationship between homophobic bullying and bullying [21,24,26,27], although they are different forms of school peer violence [24]. In relation to sex, the associations between bullying roles and use of homophobic language were different for girls and boys. For boys, these associations were large, whereas for girls they were small to moderate. This fact suggests that use of homophobic language may be more characteristic of boys who engage in bullying than for girls. However, additional research is needed to examine whether use of homophobic language is more common during bullying episodes perpetrated by boys than when perpetrated by girls [24,28].

Taking all this information into account, it is necessary to evaluate the differential characteristics of both type of bullying, and their negative effects on mental health and well-being of involved adolescents. As indicated previously, although prevalence of both types of bullying has been studied before in the previous literature, there is a lack of research analyzing and comparing prevalence and gender influence of both types of bullying in Spain. In this regard, there is a gap in the literature with respect to the evaluation and comparison of the differential effects of both types of bullying in HRQoL and mental health in involved individuals in the Spanish context. Based on previous international research, the consequences of homophobic bullying are similar to those presented by victims who have been the target of bullying behavior [23,29]. Adolescents who are victims are more likely to experience physical and mental health problems, such as high levels of anxiety, depression, suicidal ideation, stress, fear, low self-esteem and self-efficacy [14,21,29,30]. However, the impact on victims and aggressors may vary depending on the specific form of bullying experienced [29,31,32]. Although school violence and mental health issues have been shown to be associated, as previously described, there are still many gaps that need to be addressed [33], such as focusing on the comparison of specific types of harassment and bullying [34] and the need to identify a greater number of risk and protective factors to reduce the harmful effects of the different forms of peer violence [21].

The World Health Organization (WHO) states that health is not only the absence of illness, but also a state of complete physical, mental, and social well-being [35]. In this sense, there is growing interest in evaluating indicators such as the Health-Related Quality of Life (HRQoL), as they provide a holistic view of health and well-being [36]. Recent research has shown an inverse relationship between increased involvement in bullying and HRQoL in adolescents [37,38] and adults who were involved in bullying their youth [39]. Although the findings of an inverse relationship between bullying and HRQoL are consistent, being involved in some type of school violence can have a complex relationship with the psychological, physical, and social well-being of victims and aggressors. Previous studies conclude that the risk of mental health problems in adolescents varies depending on the role and typology of bullying experienced [40,41]. Hence, examining the relationship between involvement in the different forms and roles of peer violence and different dimensions of HRQoL can help to better understand the multidimensional well-being of adolescents.

For this reason, the objectives of this research were: (1) to report the frequency rates to different bullying and homophobic verbal content bullying roles in adolescents, and (2) to analyze the different patterns of Health-Related Quality of Life, anxiety and depression considering the bullying and homophobic verbal content bullying roles (uninvolved, bully, victim and bully-victim).

## 2. Materials and Methods

### 2.1. Participants and Procedure

The present study was a part of a large-scale study on school violence, victimization and wellbeing conducted in schools in the city of Alicante, Spain. Participants included a total of 1723 high school students randomly selected from five public high schools in Alicante, of which 49% were female and 51% male. Participants’ age ranged from 11 to 19 years with a mean age of 13.39 years (SD = 1.35). Inclusion criteria to take part in the study were: (a) being present in the classroom on the day of the assessment, (b) being able to read and complete the questionnaires themselves, (c) presenting an informed consent form signed by their parents allowing participation and (d) speaking and reading fluently in Spanish. Prior to collecting data from the study, parents were asked to provide written informed consent for their child to participate in the study. Students who were present on the day of data collection and accepted to participate in the study were instructed to complete an anonymous online survey in the classroom that included measures of bullying, homophobic content bullying and Health-Related Quality of Life measures. Data were collected in the classroom in presence of a research assistant from the University of Alicante during the second and third trimester of the 2016 academic year. The duration of the sessions lasted approximately 60 min. The study was approved by the Ethical Committee of the University of Alicante and by the Educational Directive Committee from Schools involved in the study (Ref. UA2015-1013).

### 2.2. Measures

#### 2.2.1. Bullying: The Illinois Bully Scale [42]

It is a self-report measure composed by 18 items with three subscales that assess: peer victimization, bully behavior and the frequency of fighting. An example of items from the peer victimization subscale includes “Other students made fun of me” or “I got hit and pushed by other students”. Examples of the bully behavior subscale include “I teased other students” or “I excluded others”. Students are asked to indicate how often in the past 30 days they have engaged in each behavior in a Likert scale that includes five response options: “Never”, “1 or 2 times”, “3 or 4 times”, “5 or 6 times”, and “7 or more times”. These response options allow the assessment of the persistence of the bullying. In the present study only the two firsts subscales (peer victimization and bully behavior) were used. Higher scores indicate more self-reported bullying behaviors. The original version had reliability values ranging from α = 0.83 to α = 0.88 for the different subscales [42]. The Spanish validation of this scale showed adequate reliability indices for both dimensions: bully behavior (α = 0.89) and peer victimization (α = 0.75) [5]. In the present study, internal consistency (Cronbach’s alpha) for each factor was acceptable: bully behavior (α = 0.91) and peer victimization (α = 0.73).

#### 2.2.2. The Homophobic Verbal Content Bullying: Homophobic Content Agent Target (HCAT) Scale [27]

It is a 10-item scale which assesses homophobic verbal content bullying including two subscales: agent and target. An example of items in the agent subscale include: “Some kids call each other names such as gay, lesbo, fag, etc. How many times in the last week did you say these things to a friend?” An example of the target subscale is “How many times in the last week did a friend call you these things”. Students respond to the items using Likert-type responses ranging from never, 1 or 2 times, 3 or 4 times, 5 or 6 times, and 7 or more times within the past week. Higher scores indicate greater frequency of homophobic verbal harassment. The original version of the HCAT has been shown to be internally consistent, α = 0.85 [35]. The Spanish version of this instrument exhibited also adequate reliability indices for both factors: victim (α = 0.78) and aggressor (α = 0.81) [43]. Cronbach’s alphas for each factor in the present study were also adequate: HCAT victim (α = 0.77) and HCAT aggressor (α = 0.81).

#### 2.2.3. Health-Related Quality of Life (HRQoL): KIDSCREEN-27 [44]

It is a questionnaire designed for the measurement of HRQoL in children and adolescents. It contains 27 items that measure HRQoL through five different dimensions: Physical Well-being (five items), Psychological Well-being (seven items), Autonomy and Parents Relations (seven items), Social Support and Peers (four items) and School Environment (four items). The response range is based on a 5-point Likert scale from ‘0’ (*never/not at all*) to ‘5’ (*always*). In the present study, Cronbach’s Alphas were adequate across all five dimensions: Physical Well-being (α = 0.83); Psychological Well-being (α = 0.87); Autonomy and Parents Relations (α = 0.82); Social Support and Peers (α = 0.77) and School Environment (α = 0.78).

#### 2.2.4. Depression: Patient Health Questionnaire (PHQ-9) [45]

It is a 9-item scale that assesses depressive symptoms. The response range varies from 0 (not at all) to 3 (nearly every day), indicating how often each item has bothered the participant over the past two weeks. Total score on the PHQ-9 range from 0 to 27. Higher scores indicate greater depressive symptomatology. The PHQ-9 is a reliable and valid measure of depression severity in general and clinical population, with a Cronbach’s alpha range of 0.86 to 0.89 in Spanish-speaking samples [46]. In the present study, Cronbach’s Alpha was adequate (α = 0.85).

#### 2.2.5. Anxiety: Generalized Anxiety Disorder-7 (GAD-7) [47]

It is a one-dimensional 7-item questionnaire designed to assess the presence of anxiety symptoms. Participants respond to each item on the scale from 0 (*not at all*) and 3 (*nearly every day*), indicating how often each item has bothered the participant over the past two weeks. Total score ranges from 0 and 21, with higher scores corresponding to greater symptomatology. The GAD-7 has been found to be a reliable and valid measure of anxiety, with a Cronbach’s Alpha of 0.93 for Spanish population [47]. In the present study, Cronbach’s Alpha was adequate (α = 0.86).

### 2.3. Data Analysis

First, participants were classified in different roles of bullying and homophobic verbal content bullying, taking into account their responses to the scales of victimization and aggression. If students responded 0 (never) or 1 (once or twice) to all the items related to aggression and victimization, they were considered uninvolved. If students responded 2 or more (three times or more) to any item about aggression and 0 or 1 to all items about victimization, they were considered bully (and vice versa for victim). If students responded 3 times or more to any items about aggression and victimization, they were considered bully-victim. This classification was done following the criterion of previous research [48]. After the role classification, differences in prevalence of participants in each role depending on sex were analyzed employing Chi-square statistics. Further, in order to identify the possible differences between roles in HRQoL, depression and anxiety, ANCOVAs were conducted controlling for the effects of sex separately for bullying and homophobic content bullying. Bonferroni correction was used in post-hoc comparisons. A value of *p* < 0.05 was considered significant in all cases. Partial eta square was used as the effect size measure. All statistical analyses were conducted using SPSS, Version 24.0 (Armonk, NY, USA).

## 3. Results

### 3.1. Frequency and Percentage for Bullying by Role

The frequency and percentages of the four assessed roles for bullying are shown in Table 1. No differences were found in the sex distribution by evaluated roles (*p* > 0.05).

### 3.2. Frequency and Percentage for Homophobic Verbal Content Bullying by Role

The frequency and percentages of the four roles assessed for homophobic verbal content bullying are presented in Table 2. Significant differences were found between groups, as more boys than girls are involved in the bully and the bully-victim role (*p* < 0.05).

### 3.3. Differences in HRQoL, Depression and Anxiety Depending on the Bullying Role

In the case of bullying, differences for role were found in all HRQoL variables: physical wellbeing [Role: F (3,1723) = 4.751, *p* = 0.003, η^2^ = 0.008], psychological wellbeing [Role: F (3,1723) = 18.808, *p* = 0.0001, η^2^ = 0.032], autonomy and parents relations [Role: F (3,1723) = 14.574, *p* = 0.0001, η^2^ = 0.025], social support and peers [Role: F (3,1723) = 10.128, *p* = 0.0001, η^2^ = 0.017], school environment [Role: F (3,1723) = 25.358, *p* = 0.0001, η^2^ = 0.042]. Sex demonstrated a significant effect only in the case of physical wellbeing [Sex: F (1,1723) = 42.807, *p* = 0.0001, η^2^ = 0.024] and psychological wellbeing [Sex: F (1,1723) = 12.582, *p* = 0.0001, η^2^ = 0.007]. Means and standard deviation for each role in HRQoL and post hoc analyses are represented in Table 3.

In the case of depression and anxiety, a significant effect of the factor Role was found in both variables [Role: F (1,1723) = 91.012, *p* = 0.0001, η^2^ = 0.137] and [Role: F (1,1723) = 48.757, *p* = 0.0001, η^2^ = 0.078] respectively. Sex had a significant effect in bot depression [Sex: F (1,1723) = 35.411, *p* = 0.0001, η^2^ = 0.020] and anxiety [Sex: F (1,1723) = 16.832, *p* = 0.0001, η^2^ = 0.010]. Means and standard deviation for each role in depression and anxiety and post hoc analyses are presented in Table 3.

### 3.4. Differences in HRQoL, Depression and Anxiety Depending on Homophobic Verbal Content Bullying Role

In the case of homophobic content bullying, differences for role were found in the following HRQoL variables: psychological wellbeing [Role: F (3,1723) = 9.984, *p* = 0.0001, η^2^ = 0.017], autonomy and parents relations [Role: F (3,1723) = 4.105, *p* = 0.006, η^2^ = 0.007], social support and peers [Role: F (3,1723) = 3.960, *p* = 0.008, η^2^ = 0.007], school environment [Role: F (3,1723) = 20.456, *p*= 0.0001, η^2^ = 0.034]. No differences were found in physical wellbeing. Sex showed a significant effect in physical [Sex: F (1,1723) = 46.180, *p* = 0.0001, η^2^ = 0.026] and psychological wellbeing [Sex: F (1,1723) = 16.911, *p* = 0.0001, η^2^ = 0.010]. Means and standard deviation for each role in HRQoL and post hoc analyses are presented in Table 4.

In the case of mental health, a significant effect of the factor Role was found for depression [Role: F (3,1723) = 53.662, *p* = 0.0001, η^2^ = 0.086] and anxiety [Role: F (3,1723) = 34.790, *p* = 0.0001, η^2^ = 0.057]. In both cases, the factor sex had a significant effect for depression [Sex: F (1,1723) = 52.118, *p* = 0.0001, η^2^ = 0.029] and anxiety [Sex: F (1,1723) = 27.608, *p* = 0.0001, η^2^ = 0.016]. Means and standard deviation for each role in depression and anxiety and post hoc analyses are presented in Table 4.

## 4. Discussion

The present study aimed to identify the prevalence of bullying and homophobic verbal content bullying in a sample of Spanish adolescents, considering the specific frequency of adolescents involved in different roles in each case. Furthermore, differences in HRQoL and mental health, based on the role in which the adolescent was involved, were also analyzed. Considering the obtained results, prevalence of bullying is in accordance to previous research conducted in Spanish samples [3,4,5]. However, in the case of homophobic verbal content bullying, as far as we know, this is one of the first studies conducted in Spain assessing the prevalence of this explicit type of bullying through a specific evaluation instrument originally designed to the analysis of this type of harassment for all individuals, independently of the sexual orientation. Employing other type of measures, some studies have been recently conducted in Spain, such as the Rodríguez-Hidalgo’s and colleagues one [30]. In this study, in which authors employed an adaptation of a previous questionnaire oriented to the evaluation of traditional bullying, the prevalence of victims of homophobic bullying was of 23%. In the present study, only the 9.8% of adolescents were identified as victims, a prevalence much lower in comparison to the previous study. Differences in the prevalence between studies could be based on the differential age range of the sample considered and the employed instruments. In this regard, it has been demonstrated that bullying prevalence increases as age of adolescents increases, being more prevalent in advanced school stages [2,3]. Furthermore, in the present research it has been employed a specific questionnaire originally designed for the homophobic verbal content bullying, probably being more reliable for the analysis of this type of bullying. In addition, the study conducted by Rodríguez-Hidalgo and colleagues [30] evaluated the presence of homophobic bullying during the last 2 months, while in the present study it has been assessed during the last week.

With regard to the differences found in prevalence between both types of bullying, based on the obtained results, there is more percentage of victims in the case of homophobic verbal content bullying than in traditional bullying. The mechanism involved in such high prevalence of victims in this type of bullying could respond to a normalization of homophobic verbalizations in school environment. In a previous study which analyzed microaggressions and harassment of LGBTQ youth in schools, 43% of school psychologist participating in the study declared to have listened regularly homophobic epithets, such as “that’s so gay” or the use of the term “faggot” in the school context [49]. However, when students are asked about this issue, the prevalence is much higher, as 90% of them stated to have listened one or more homophobic comments in class, even in presence of teachers [50]. Hence, in the previous cited study, 45% of school psychologists reported hearing other teachers employing this vocabulary [49]. Moreover, some adolescents reported the employment of this type of homophobic language in a friendly context, jokingly, fundamentally between boys’ interactions, with no harassment intentions [51]. These results reinforce the idea of a homophobic language normalization in the school context. Attending to these results, the higher frequency of homophobic expressions and vocabulary seems to be a plausible mechanism that could explain the higher prevalence of victimization regarding homophobic verbal content bullying.

Considering sex differences in roles prevalence, only in the case of homophobic verbal content bullying, differences were found, being boys more involved in the bully and the bully-victim roles, in comparison with girls. Traditionally, several studies have demonstrated that boys are more involved in homophobic bullying, in both roles of aggression and victimization [27,52,53]. As indicated by previous research, males seem to report higher homophobia levels, as they express more frequently negative attitudes and behaviors towards members of the LGBT group in comparison with females [27,52,53]. This fact, together with the idea that boys are more frequently the target of homophobic aggressions, could explain this high prevalence in the bully-victim role.

With regard to HRQoL, the uninvolved individuals in both types of bullying are those participants who exhibited the better results in HRQoL. Contrary, those most affected are those bully-victim adolescents. These results are similar to those obtained in recent studies in which differences in HRQoL by role were assessed [27,54,55]. When single roles were evaluated, those adolescents who suffer bullying victimization exhibited poorer HRQoL than aggressors [54]. However, based on our results, being involved in both aggression and victimization entails the worst results for HRQoL, similar to previous studies [54,55]. It has been suggested that aggressive victims are at high risk of health and quality of life disruption due to their deep involvement in bullying behavior in schools [55]. Fundamentally, based on classical hypothesis, bully-victims bully in response to being bullied [56,57], and hence, assume the negative consequences of both roles. Although the negative consequences for both bullying and homophobic verbal content bullying on HRQoL and mental health are similar for both types of bullying, based on the obtained results, it seems that the traditional bullying has a higher impact on these variables, fundamentally in the case of physical wellbeing. The fact that traditional bullying involves physical aggressions and the homophobic verbal content bullying only verbal aggressions, may explain why physical wellbeing is more deteriorated in the former.

The obtained results for mental health variables are similar, although higher differences between roles were found. This seems to indicate that anxiety and depression are more affected by both types of bullying than general HRQoL. Previous studies have demonstrated how bullying and homophobic bullying are highly related to mental health deterioration in youth [53,58], fundamentally if the victimization becomes chronic [59]. As in the case of HRQoL, it seems that traditional bullying has a higher negative impact in mental health than homophobic verbal content bullying, attending the levels of depression and anxiety symptoms.

To the best of our knowledge, this is one of the first studies conducted in Spain that has assessed bullying and homophobic verbal content bullying and their relationship with HRQoL, depression and anxiety in adolescents. Although the obtained results contribute significantly to the knowledge about the negative consequences of different types of bullying on health, quality of life and wellbeing of adolescents, some limitations of the study should be noted. First, the cross-sectional design of the study does not allow establishing causal relationship between variables. Second, the data was obtained through the employment of self-reported measures, and, although it has been demonstrated that they are reliable and valid measures, the results could be influenced by the subjective perception of participants. Thirdly, the instruments employed to assess bullying and homophobic verbal content bullying differed in the time period they evaluated (one month and one week). Considering that we decide to maintain the original instruction in both questionnaires, the comparison of percentages should be considered carefully. Future studies are needed to explore the link between homophobic verbal content bullying and traditional bullying and the overlapping of both. In any case, several strengths of the study should be taken into account. The sample of the study was sufficiently large and representative to assure the generalization of the obtained results. Unlike previous research, it has been employed a questionnaire originally developed and validated for the evaluation of homophobic content verbal bullying, which increase the reliability of the obtained results regarding the prevalence of this type of bullying.

## 5. Conclusions

From the results obtained in the present study several clinical and educative implications could be derived. From a clinical perspective, it has been pointed out that both forms of bullying, traditional and the homophobic one, have a negative impact on HRQoL and mental health of adolescents. Hence, this group of adolescents could be at high risk for a significant health deterioration, something that should be taken into account by educators and clinicians, in order to develop preventive and intervention strategies, mainly oriented to this high-risk group. From an educative viewpoint, the present study highlights the necessity of the promotion of a school context based on acceptation and inclusion, eradicating any form of discrimination based on the sexual orientation of adolescents. As has been showed the previous results, levels of homophobic bullying in Spain stills being high, entailing a main issue that educators and school counselors should face with extremely urgency. Future studies should analyze the differential effects on HRQoL and mental health of other highly prevalent types of bullying, such as cyberbullying. As it can be identified in the present study, diverse typologies of bullying could have a different impact on health and wellbeing of adolescents, but future research should demonstrate this fact including new forms of bullying. Moreover, new studies should be developed in order to replicate these results in other age groups or even other educational stages and to identify the possible effect of sexual orientation in the obtained results.

## Figures and Tables

**Table 1:** Frequency and Percentage of Bullying by Role.

Role	Total	Boy *n* (%)	Girl *n* (%)	*χ* ^2^	*p*
Uninvolved	710 (41.2%)	341 (48.03%)	369 (51.97%)	4.374	0.224
Bully	550 (31.9%)	294 (53.5%)	256 (46.5%)		
Victim	141 (8.2%)	74 (52.5%)	67 (47.5%)		
Bully-Victim	322 (18.7%)	170 (52.8%)	152 (47.2%)		

**Table 2:** Frequency and Percentage of Homophobic Verbal Content Bullying by Role.

Role	Total	Boy *n* (%)	Girl *n* (%)	*χ* ^2^	*p*
Uninvolved	1046 (60.70%)	472 (45.1%)	574 (54.9%)	62.884	0.0001
Bully	241 (13.99%)	138 (57.3%)	103 (42.7%)		
Victim	169 (9.82%)	79 (46.7%)	90 (53.3%)		
Bully-Victim	267 (15.49%)	190 (71.2%)	77 (28.8%)		

**Table 3:** Means and Standard Deviations in HRQoL Dimensions, Depression and Anxiety for Each Bullying Role.

Variables	Uninvolved (*n* = 710)	Bully (*n* = 550)	Victim (*n* = 141)	Bully-Victim (*n* = 322)	Post-Hoc
	M (SD)	M (SD)	M (SD)	M (SD)	
KIDSCREEN					
Physical Well-being	19.15 (3.81)	18.94 (3.82)	19.17 (3.95)	18.25 (4.15)	U > BV
Psychological Well-being	29.48 (4.66)	28.69 (4.73)	27.89 (5.37)	27.23 (4.66)	U > B, V, BV B > BV
Autonomy and Parents Relations	30.02 (4.48)	29.15 (4.59)	29.04 (4.58)	29.03 (4.88)	U > B, BV B > BV
Social Support and Peers	17.88 (2.43)	17.73 (2.16)	17.06 (3.16)	17.09 (2.72)	U > V, BV B > V, BV
School Environment	16.24 (2.73)	15.48 (2.90)	15.51 (3.31)	14.58 (2.98)	U > B, V, BV B, V > BV
Depression	2.85 (3.47)	4.09 (4.06)	5.39 (4.72)	7.25 (5.22)	U < B, V, BV B < V, BV V < BV
Anxiety	2.94 (3.68)	3.75 (3.60)	4.85 (3.91)	5.90 (4.47)	U < B, V, BV B < V, BV V < BV

Note: M = Mean, SD = Standard Deviation; U = Uninvolved; B = Bully, V = Victim; BV = Bully-Victim.

**Table 4:** Means and Standard Deviations in HRQoL Dimensions, Depression and Anxiety for Each Homophobic Verbal Content Bullying Role.

Variables	Uninvolved (*n* = 1046)	Bully (*n* = 241)	Victim (*n* = 169)	Bully-Victim (*n* = 267)	Post-Hoc
	M (SD)	M (SD)	M (SD)	M (SD)	
KIDSCREEN					
Physical Well-being	19.02 (3.79)	18.76 (4.08)	18.85 (4.22)	18.70 (3.96)	-
Psychological Well-being	29.12 (4.84)	28.19 (5.06)	28.07 (4.64)	27.78 (4.39)	U > B, V, BV
Autonomy and Parents Relations	29.55 (4.75)	29.21 (4.50)	28.91 (4.65)	28.60 (4.31)	U > BV
Social Support and Peers	17.78 (2.48)	17.47 (2.48)	17.40 (2.51)	17.25 (2.50)	U > BV
School Environment	15.98 (2.84)	15.48 (2.83)	15.44 (3.07)	14.47 (3.04)	BV < U, B, V
Depression	3.41 (3.80)	4.58 (4.36)	5.81 (5.07)	6.42 (5.32)	U < B, V, BV B < BV
Anxiety	3.24 (3.67)	4.36 (3.95)	5.06 (4.15)	5.41 (4.44)	U < B, V, BV B < BV

Note: M = Mean, SD = Standard Deviation; U = Uninvolved; B = Bully, V = Victim; BV = Bully-Victim.
